# Supplementary material for: Identification of photoperiod-induced specific miRNAs in the adrenal glands of Sunite sheep (Ovis aries)
Source: Front Vet Sci. 2022 Jul 22;9:888207. doi: 10.3389/fvets.2022.888207 (PMC9354845; doi:10.3389/fvets.2022.888207)
Supplement: Supplementary file 1 [file Data_Sheet_1.ZIP › Supplementary file/miRDeep2 file.zip!/miRDeep2 file/Know_Structure/SP42A3/oar-mir-21.pdf]

miRBase precursor : oar-mir-21
Total read count : 215633
oar-miR-21 read count : 215622
remaining reads : 11

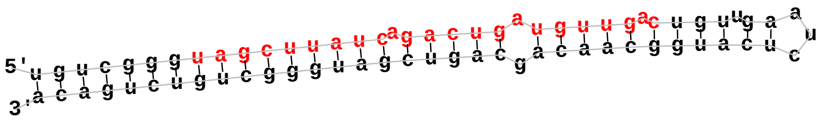

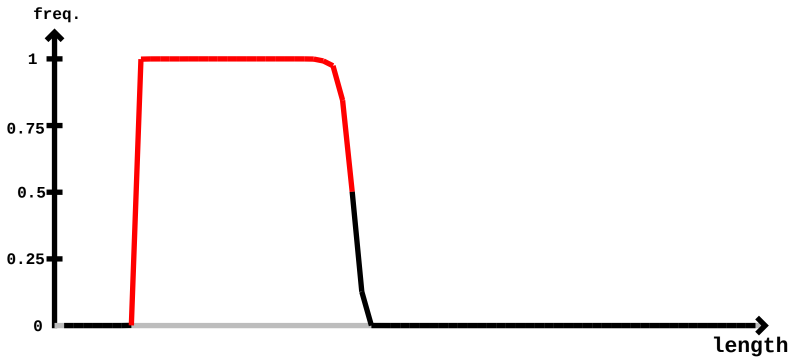

oar-miR-21

```
5'- ugucggguagcuuaucagacugauguugacuguugaaucucauggcaacagcagucgaugggcugucugaca   -3'   exp
    ((((((((((((((((.(((((.(((((.((((.((...))))))))))).)))))))))))))))))))))   reads   mm   sample
    ......guagcuuaucagacugaugu..............................................   1       0    seq
    ......guagcuuaucagacugauguu.............................................   8       0    seq
    ......guagcuuaucagacugauguug............................................   53      0    seq
    ......guagcuuaucagacugauguuga...........................................   68      0    seq
    ......guagcuuaucagacugauguugac..........................................   16      0    seq
    ......guagcuuaucagacugauguugacu.........................................   5       0    seq
    .......uagcuuaucagacugau................................................   11      0    seq
    .......uagcuuaucagacugaug...............................................   124     0    seq
    .......uagcuuaucagacugaugu..............................................   1538    0    seq
    .......uagcuuaucagacugauguu.............................................   3887    0    seq
    .......uagcuuaucagacugauguug............................................   27803   0    seq
    .......uagcuuaucagacugauguuga...........................................   73865   0    seq
    .......uagcuuaucagacugauguugac..........................................   80693   0    seq
    .......uagcuuaucagacugauguugacu.........................................   27313   0    seq
    .......uagcuuaucagacugauguugacug........................................   3       0    seq
    .......uagcuuaucagacugauguugacugu.......................................   18      0    seq
    .......uagcuuaucagacugauguugacuguu......................................   4       0    seq
    .......uagcuuaucagacugauguugacuguugaau..................................   2       0    seq
    ........agcuuaucagacugaugu..............................................   1       0    seq
    ........agcuuaucagacugauguu.............................................   2       0    seq
    ........agcuuaucagacugauguug............................................   18      0    seq
    ........agcuuaucagacugauguuga...........................................   62      0    seq
    ........agcuuaucagacugauguugac..........................................   57      0    seq
    ........agcuuaucagacugauguugacu.........................................   35      0    seq
    .........gcuuaucagacugauguu.............................................   1       0    seq
    .........gcuuaucagacugauguug............................................   4       0    seq
    .........gcuuaucagacugauguuga...........................................   13      0    seq
    .........gcuuaucagacugauguugac..........................................   8       0    seq
    .........gcuuaucagacugauguugacu.........................................   4       0    seq
    .........gcuuaucagacugauguugacuguugaauc.................................   1       0    seq
    ...........uuaucagacugauguuU............................................   1       1    seq
    ...........uuaucagacugauguugaU..........................................   2       1    seq
    ............uaucagacugauguuUa...........................................   1       1    seq
    ............uCucagacugauguuga...........................................   1       1    seq
    ............uaucagacugauguuga...........................................   1       0    seq
```

oar-miR-21

```
ugucggguagcuuaucagacugauguugacuguugaaucucauggcaacagcagucgaugggcugucugaca
............uaucagacugauguugac.........................................    1    0    seq
.............................................caacagcagucgaugggcu........    1    0    seq
.............................................caacagcagucgaugggcug.......    1    0    seq
.............................................caacagcagucgaugggcugu......    2    0    seq
.............................................caacagcagucgaugggcuguc.....    4    0    seq
```
